# Electronic differential control based on speed and optimum slip ratio estimation for all-electric vehicles with in-wheel motors

**DOI:** 10.1371/journal.pone.0325125

**Published:** 2025-06-02

**Authors:** Huan Liu

**Affiliations:** School of Electronics and Communication Engineering, Lanzhou University of Arts and Science, Lanzhou, Gansu, China; National University of Singapore, SINGAPORE

## Abstract

Vehicle 2-degree of freedom (DOF) kinematic and dynamic models are derived. The former, which uses fixed parameters, is often used for speed-based electronic differential control, but this method does not yield accurate results under varying running situations. In contrast, the latter, which depends on the tire adhesion limit to produce tire saturation force, is typically adopted for torque-based electronic differential control. However, this method also faces many difficulties in real-time implementation, and its theoretical maturity is not strong. To combine the advantages of speed-based electronic differential control and torque-based electronic differential control, this paper focuses on speed and optimum slip ratio as key factors. Additionally, to address the difficulties associated with nonlinear modeling by leveraging the simplicity of linear modeling in design and implementation, this study presents an electronic differential control based on speed and optimum slip ratio estimation for all-electric vehicles with in-wheel motors. It aims to maintain the maneuvering ability of the driver at the maximum adhesion limit. Even when the two driving wheels are subjected to uneven external disturbances from the road surface, they maintain a synchronous speed when driving on a straight line or a differential speed when turning. Simulation validation confirms that the proposed method enhances safety for in-wheel motor electric vehicles in urban scenarios involving adverse weather conditions (e.g., rain, snow, ice) and aggressive lane-changing maneuvers. Experimental validation confirms the static performance of the motor controller and the differential control capability of the two drive wheels. These findings lay a foundation for improving extreme-condition adaptability through three future directions: adaptive tire dynamics integration, hierarchical energy-stability control architectures, and real-time deployment validated via hardware-in-the-loop testing.

## 1 Introduction

The pursuit of electric vehicles (EVs) as a solution to environmental and energy challenges has spurred extensive research and development efforts focused on enhancing the practicality of electric vehicle technology [1, 2]; ensuring battery sustainability [3]; optimizing charging and replacement systems [4]; implementing smart energy solutions for electric vehicles [5, 6]; as well as exploring hydrogen energy and fuel cells which remain highly relevant topics [7, 8].

**Fig 1:**
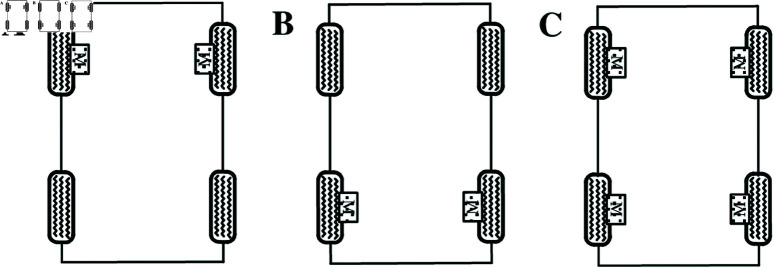
Driveline configurations for EVs with in-wheel motors. A: Front-wheeled-driven. B: Rear-wheeled-driven. C: Four-wheeled-driven. https://doi.org/10.6084/m9.figshare.28785026.

Electric vehicles with in-wheel motors are driven by two or four wheels attached to the EVs. The in-wheel motors are directly linked to each driving wheel. Fig 1 shows a schematic of the driveline configurations, which are the subjects of this study. This study focuses mainly on rear-wheeled-driven all-electric vehicles.

When a vehicle turns a corner or runs on an uneven road surface, the driving-wheel velocity on both sides will differ, causing differential control problem. The two main approaches currently employed for realizing electronic differential control are special motor design based on adaptive differential and vehicle controller design based on speed or torque, supplemented with driving-wheel velocity and torque.

### 1.1 Special motor design based on adaptive differential

An adaptive differential motor is designed following the principle of mechanical differential and has an automatic differential function. Its main feature is a two-rotor structural design, which is usually called a dual-rotor motor. Unlike traditional motors featuring a single stator, rotor, and mechanical port, a dual-rotor motor has two mechanical shafts that can transfer energy independently, facilitating the transfer of energy between two separate channels. Although the dual-rotor motor offers reduced volume and weight, it also has a higher power density with significantly improved efficiency. Moreover, it can meet the multi-power source coupling output of electromechanical energy, conserve energy, protect the environment, and have various application prospects, particularly in the EV industry. According to different structures and principles, dual-rotor motors can be divided into four types: dual-rotor synchronous–asynchronous motors, squirrel-cage dual-rotor motors, contra-dual-rotor permanent magnet motors, and permanent magnet brushless dual-rotor motors [9]. A dual independent rotor axial flux induction motor (DIR-AFIM) with two degrees of freedom was proposed in [10]. The motor is equipped with two independent rotors, each capable of providing propulsion for the electric vehicle and functioning as a differential, thereby eliminating the need for mechanical differentials. However, its application is limited to small vehicles accommodating one or two occupants.

### 1.2 Electronic differential control based on speed

The vehicle 2-degree of freedom (DOF) kinematic model with fixed parameters is often adopted for speed-based electronic differential control. When turning, the inner- and outer-wheel velocities can be obtained using the Ackermann’s steering principle. The steering angle is used to calculate the wheel velocity. An electronic differential strategy based on sliding mode control was devised for three-phase four-switch inverters, enabling torque distribution between wheels via dynamic switching frequency adjustment [11]. For velocity estimation, a generalized neural network algorithm incorporating vehicle aerodynamics has been employed to predict vehicle velocity during steering maneuvers [12], while a sensorless velocity measurement method leveraging armature current and terminal voltage parameters was proposed to enhance the accuracy of driving-wheel velocity [13]. In synchronization control, a general fictitious master synchronization framework exhibited superior disturbance rejection and transient response capabilities [14]. Concerning differential configurations, three electronic differential (ED) architectures for four-wheel-drive electric vehicles—front-wheeled-driven ED, rear-wheeled-driven ED, and four-wheeled-driven ED—were comprehensively compared through normal cornering and circling maneuver tests [15]. Energy-managed longitudinal acceleration control improved operational efficiency but demonstrated limited adaptability on steep slopes and uneven terrain [16]. For motion tracking, a vehicle velocity estimation methodology utilizing driving-wheel angular velocity and torque data provided alternatives to mechanical differentials [17, 18]. In low-speed torque allocation, an active disturbance rejection controller dynamically adjusted inner-outer wheel torque based on ideal angular velocity references [19]. At high speeds, a quadratic programming optimization approach was introduced for differential control, although computational complexity restricted real-time performance [20]. To enhance robustness, a slip ratio-based constant sliding mode controller was utilized to optimize torque distribution through driving-wheel slip feedback [21]. Meanwhile, a threshold-controlled electronic differential, which considered the sideslip angle, centripetal force, and axle load transfer, improved adaptability in complex steering scenarios [22]. Although an algorithm integrating dynamic parameter thresholds combined sliding mode control with quadratic programming optimization, its multi-strategy coordination led to increased system complexity [23]. Recent advancements in four-wheel independent hub motor systems have adopted Ackermann steering models for initial speed allocation and vehicle dynamics models for wheel speed correction; however, further improvements are still required for complex road conditions [24].

### 1.3 Electronic differential control based on torque

The vehicle 2-DOF dynamic model, which depends on the tire adhesion limit to produce a tire saturation force, is often adopted for torque-based electronic differential control. All driving wheels can be controlled independently based on the driving force control. Early research [25] proposed an optimum slip ratio estimation method that relied on vehicle velocity but was unsuitable for four-wheel-drive vehicles. Subsequent work [26] presented a method to estimate the slip ratio based on the velocity and torque of the driving wheel without detecting the vehicle velocity, but the performance was poor for non-linear models. For planetary rover applications, two distinct estimation methods emerged: one combined terrain mechanics and a lunar rover dynamic model using extended Kalman filter technology [27], and the other was based on wheel-terrain images using only visual means [28]. Road adhesion coefficient estimation has been addressed through fading memory unscented Kalman filtering [29], while hierarchical control architectures have been implemented for stability across varying adhesion conditions, but some problems exist in switching between the two modes [30]. Recent developments include yaw moment control using sliding mode techniques with quadratic wheel load optimization [31], slip ratio estimation incorporating road type classification and tire deflection rates [32], and variable structure and sliding mode methods for wheel slip control [33]. However, these methods show limitations in handling μ-split road surfaces or complex motion scenarios [34]. Adaptive differential control strategies have evolved to address torque distribution challenges. Initial implementations neglected critical tire-road adhesion constraints [35, 36], while later approaches integrated fuzzy logic for four-wheel torque allocation to enhance lateral stability [37]. Model predictive control frameworks have been applied to coordinate traction and braking forces while addressing traditional stability control inaccuracies [38]. Through the implementation of a real-time nonlinear model predictive control strategy, the direct yaw moment control challenge in distributed drive electric vehicles has been effectively addressed. This significantly enhances vehicle stability while simultaneously reducing computational complexity [39]. To ensure tracking convergence within the DYC system, a Lyapunov-based nonlinear model predictive control strategy is incorporated. This approach markedly improves the overall dynamic performance and control precision of distributed drive electric vehicles [40]. For electric racing vehicles, simplified estimation models have been enhanced through sideslip angle prediction [41], though battery dynamics and suspension effects remain underexplored. Advanced torque distribution strategies now consider vertical load transfer and steering conditions through scenario-specific torque allocation [42]. Multi-objective optimization approaches aim to balance stability during sudden maneuvers with energy efficiency [43], while adaptive differential steering strategies enable configuration-variable unmanned vehicles to handle complex tasks through modified sliding mode control [44]. Current hierarchical control systems combine yaw moment determination with slip ratio regulation [45], though high-speed lateral stability remains challenging. Latest innovations incorporate operational mode recognition into adhesion coefficient identification for predictive slip ratio control [46], representing significant progress in adaptive torque distribution systems.

Currently, the application of special motor designs based on adaptive differential faces limitations and challenges owing to inherent defects. Consequently, designing a vehicle controller based on speed, torque, or a combination of diverse strategies has emerged as a more practical and feasible approach. However, speed- or torque-based electronic differential control has its limitations. The traditional static speed allocation method derived from the Ackermann-Jeantand model has two significant drawbacks: first, it neglects tire dynamic characteristics, such as the influence of centrifugal force; second, there exists a degree-of-freedom constraint between the four-wheel velocity and vehicle velocity, which compromises dynamic coordination capabilities. While the torque distribution strategy based on vehicle dynamics can partially mitigate tire drag, it still reveals dual contradictions under complex road conditions: on the one hand, abrupt fluctuations in load torque result in an exponential increase in control complexity; on the other hand, the independent wheel control mechanism lacks kinematic coordination, thereby exacerbating the risk of system oscillation. Consequently, it is imperative to develop a more efficient control strategy that leverages the advantages of the two is crucial. The main contributions of this study are as follows:

- Design a more practical and feasible vehicle controller by integrating two motor controllers and a differential controller into a unified system, utilizing speed, torque, or a combination of strategies.
- Use the simplicity of linear modeling in design and implementation to solve the difficulties with nonlinear modeling for optimum slip ratio estimation.
- This study presents an electronic differential control based on speed and optimum slip ratio estimation.

The remainder of this paper is organized as follows: Sect 2 provides an in-depth analysis of the vehicle 2-DOF kinematic model and vehicle 2-DOF dynamic model. Sect 3 elaborates on the estimation method for the optimum slip ratio and defines the precise process for identifying the optimum slip ratio. Sect 4 presents the electronic differential control based on speed and optimum slip ratio estimation, which controls the driving wheel slip ratio within the optimal range to enhance vehicle safety performance. Sects 5 and 6 present the analyses of simulation results and experimental results, respectively. Finally, Sect 7 summarizes the research content of this paper and proposes potential directions for improvement and future expansion ideas.

## 2 Vehicle model

### 2.1 Vehicle 2-DOF kinematic model

**Fig 2:**
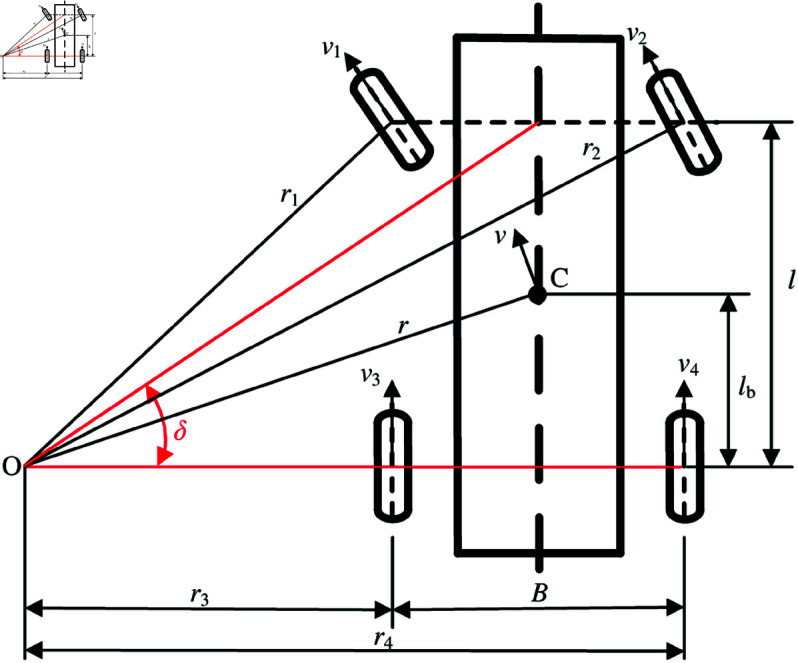
Vehicle 2-DOF kinematic model. https://doi.org/10.6084/m9.figshare.28787606.

The vehicle 2-DOF kinematic model uses the Ackermann–Jeantand model (Fig 2). O is the center of turn and C is the center of gravity. The main assumptions of the vehicle 2-DOF kinematic model in this analysis are as follows:

(1) The chassis is considered rigid.

(2) The friction force between the road and driving wheel is sufficient to prevent slipping.

(3) When the vehicle is driving, the tire is always in good contact with the ground.

(4) Wheel cornering stiffness is large enough and proportional to the maximum road attachment coefficient.

Rear wheel steering radii using basic trigonometry can be calculated by Eq 1.

$$\left\{ \begin{array}{c} r_{3}=l\cot\delta -\frac{1}{2}B\\ r_{4}=l\cot\delta +\frac{1}{2}B \end{array}\right.$$ (1)

Rear wheel velocities can be expressed as

$$\frac{v_{3}}{r_{3}}=\frac{v_{4}}{r_{4}}=\frac{v}{r},$$ (2)

where *r*=$\sqrt{l_{\text{b}}^{2}+(l\cot\delta {)}^{2}}$.

Rear wheel velocities can be expressed as

$$\left\{ \begin{array}{c} v_{3}=\frac{v}{\sqrt{l_{\text{b}}^{2}+(l\cot\delta {)}^{2}}}(l\cot\delta -\frac{B}{2})\\ v_{4}=\frac{v}{\sqrt{l_{\text{b}}^{2}+(l\cot\delta {)}^{2}}}(l\cot\delta +\frac{B}{2}) \end{array}\right.$$ (3)

Rear wheel angular velocity references can be expressed as

$$\left\{ \begin{array}{cc} {\omega_{3}}^{*}=\frac{v(l\cot\delta -B/2)}{r_{\text{W}}\sqrt{l_{\text{b}}^{2}+(l\cot\delta {)}^{2}}}\\ & ,\\ {\omega_{4}}^{*}=\frac{v(l\cot\delta +B/2)}{r_{\text{W}}\sqrt{l_{\text{b}}^{2}+(l\cot\delta {)}^{2}}} \end{array}\right.$$ (4)

where $\omega_{3}^{*}$ and $\omega_{4}^{*}$ are rear driving-wheel angular velocity references, and $r_{\text{W}}$ is the driving-wheel radius.

### 2.2 Vehicle 2-DOF dynamic model

**Fig 3:**
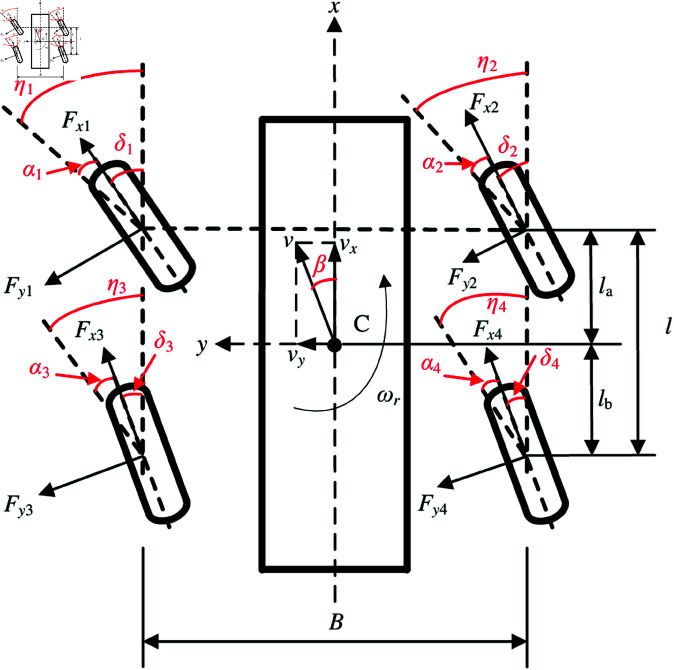
Vehicle 2-DOF dynamic model. https://doi.org/10.6084/m9.figshare.28787648.

The vehicle 2-DOF dynamic model is shown in Fig 3. An orthogonal vehicle coordinate system Gxyz is established. The origin of the orthogonal vehicle coordinate system G is coincident with the center of gravity C. The Gx-axis is longitudinal and points toward the vehicle direction. The Gy-axis is transverse and perpendicular to the Gx-axis, and is directed to the left of the driver. The Gz-axis is perpendicular to the other two and is directed upward. If the vehicle movement along the Gz-axis is ignored, the main assumptions in this analysis for the vehicle 2-DOF dynamic model are as follows:

(1) The two in-wheel motor torques and friction forces are the same in the left and right tires.

(2) The steering angle and the front-wheel steering angle have the linear form, and $\delta_{1}$=$\delta_{2}$.

(3) The vehicle driveline system is ideal, and its elastic damping is neglected.

(4) The suspension system is neglected to simplify the analysis.

(5) The steering wheel swing vibration is ignored.

The equations can be expressed as

$$m_{\text{c}}({\dot{v}}_{y}+v_{x}\omega_{\text{r}})=Y_{1}+Y_{2}+Y_{3}+Y_{4},$$ (5)

$$J_{\text{z}}{\dot{\omega }}_{\text{r}}=l_{\text{a}}(Y_{1}+Y_{2})-l_{\text{b}}(Y_{3}+Y_{4})-B(X_{1}+X_{3})/2+B(X_{2}+X_{4})/2,$$ (6)

where

$X_{i}=F_{xi}\cos\delta_{i}-F_{yi}\sin\delta_{i}\approx F_{xi}-F_{yi}\delta_{i}$, *i*=1, 2, 3, 4;

$Y_{i}=F_{xi}\sin\delta_{i}+F_{yi}\cos\delta_{i}\approx F_{xi}\delta_{i}+F_{yi}$, *i*=1, 2, 3, 4.

Wheel longitudinal force can be expressed as Eq 7.

$$F_{xi}=\mu_{i}F_{zi}$$ (7)

Wheel lateral forces can be expressed as Eq 8.

$$F_{yi}=K_{\text{a}i}\alpha_{i}$$ (8)

Let the front-wheel steering angle be $\delta_{\text{f}}$, and the rear-wheel steering angle be $\delta_{\text{r}}$, then Eq 5 and Eq 6 can be rewritten as

$$m_{\text{c}}({\dot{v}}_{y}+v_{x}\omega_{\text{r}})=\frac{K_{\text{af}}+K_{\text{ar}}}{v_{x}}v_{y}+\frac{l_{\text{a}}K_{\text{af}}-l_{\text{b}}K_{\text{ar}}}{v_{x}}\omega_{\text{r}}-K_{\text{af}}\delta_{\text{f}},$$ (9)

$$J_{\text{z}}{\dot{\omega }}_{\text{r}}=\frac{l_{\text{a}}K_{\text{af}}-l_{\text{b}}K_{\text{ar}}}{v_{x}}v_{y}+\frac{l_{\text{a}}^{2}K_{\text{af}}+l_{\text{b}}^{2}K_{\text{ar}}}{v_{x}}\omega_{\text{r}}-l_{\text{a}}K_{\text{af}}\delta_{\text{f}}+\frac{B}{2}(F_{x4}-F_{x3}),$$ (10)

where $K_{\text{af}}$ is the front-wheel cornering stiffness, and $K_{\text{a}r}$ is the rear-wheel cornering stiffness.

The rear wheel sideslip angles can be expressed as Eq 11.

$$\left\{ \begin{array}{cc} \alpha_{3}=-\delta_{\text{r}}+\arctan\left(\frac{v_{y}-l_{\text{b}}\omega_{\text{r}}}{v_{x}-\frac{B}{2}\omega_{\text{r}}}\right)\approx -\delta_{\text{r}}+\frac{v_{y}-l_{\text{b}}\omega_{\text{r}}}{v_{x}}\\ & \left(\left|v_{x}\right|\gg \frac{B}{2}\omega_{\text{r}}\right)\\ \alpha_{4}=-\delta_{\text{r}}+\arctan\left(\frac{v_{y}-l_{\text{b}}\omega_{\text{r}}}{v_{x}+\frac{B}{2}\omega_{\text{r}}}\right)\approx -\delta_{\text{r}}+\frac{v_{y}-l_{\text{b}}\omega_{\text{r}}}{v_{x}} \end{array}\right.$$ (11)

The rear wheel velocities can be expressed as Eq 12.

$$\left\{ \begin{array}{c} v_{3}=\sqrt{(v_{x}-B\omega_{\text{r}}/2{)}^{2}+(v_{y}-l_{\text{b}}\omega_{\text{r}}{)}^{2}}\cos\alpha_{3}\\ v_{4}=\sqrt{(v_{x}+B\omega_{\text{r}}/2{)}^{2}+(v_{y}-l_{\text{b}}\omega_{\text{r}}{)}^{2}}\cos\alpha_{4} \end{array}\right.$$ (12)

The centripetal force can be expressed as

$$F_{\text{c}}=m_{\text{c}}\dot{v}(\omega_{\text{r}}+\dot{\beta }),$$ (13)

where $\beta =\arctan(v_{y}/v_{x})$, $v=\sqrt{v_{x}^{2}+v_{y}^{2}}$.

The rear wheel vertical loads can be expressed as Eq 14.

$$\left\{ \begin{array}{c} F_{z3}=\frac{l_{\text{a}}}{2l}(m_{\text{c}}g-\frac{2hF_{\text{c}}}{B})\\ F_{z4}=\frac{l_{\text{a}}}{2l}(m_{\text{c}}g+\frac{2hF_{\text{c}}}{B}) \end{array}\right.$$ (14)

## 3 Optimum slip ratio estimation

**Fig 4:**
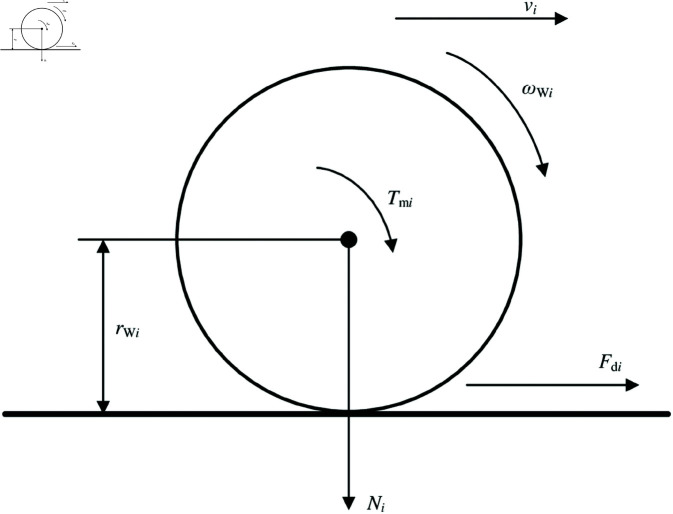
Driving-wheel dynamic model. https://doi.org/10.6084/m9.figshare.28787690.

Ignoring air resistance, pitching, and rolling, a simple single-wheel vehicle model is usually adopted to deal with the slip phenomenon. Fig 4 shows the driving-wheel dynamic model.

As can be seen from Fig 4, the rotational motion of driving wheel can be expressed as Eq 15.

$$J_{\text{W}i}\frac{d\omega_{\text{W}i}}{dt}=T_{\text{m}i}-F_{\text{d}i}\cdot r_{\text{W}i}$$ (15)

The wheel attachment coefficient can be expressed as Eq 16.

$$\mu_{i}(\lambda_{i})=\frac{F_{\text{d}i}}{N_{i}}$$ (16)

The relationship between the wheel attachment coefficient and the slip ratio can be expressed as Eq 17

$$\mu_{i}(\lambda_{i})=\frac{1}{N_{i}r_{\text{W}i}}\left(T_{\text{m}i}-J_{\text{W}i}\frac{d\omega_{\text{W}i}}{dt}\right)$$ (17)

When the vehicle load is certain, the driving wheel’s vertical force and radius can be considered a large inertia system. Therefore, *N*_*i*_ and $r_{\text{W}i}$ can be regarded as constant. Thanks to in-wheel motor technologies, the driving-wheel angular velocity can be measured conveniently. The driving-wheel driving torque can also be easily estimated. Notably, Eq 17 can be used to determine the current value of the wheel attachment coefficient between the tire and road. This means that road conditions should be estimated relatively quickly.

**Fig 5:**
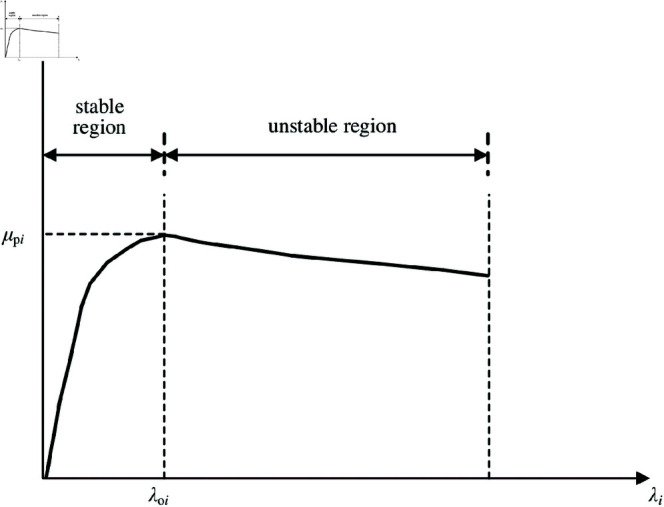
μ–λ curve. https://doi.org/10.6084/m9.figshare.28787735.

When an EV is driven on a slippery or snowy road, the driving-wheel slips, which can cause serious problems, such as spin or drift-out. The friction force arising from tire deflection changes substantially depending on the wheel attachment coefficient between the tire and road. Although the wheel attachment coefficient relies heavily on vehicle speed, tire characteristics (aging, wear, compound, etc.), and road surface conditions (tarmac, gravel, icy, dry, etc.), it can be considered a function of the slip ratio between the two contact surfaces (Fig 5). In the stable region, the driving-wheel slip ratio is relatively small, and the wheel attachment coefficient proportionally increases the driving-wheel slip ratio. Furthermore, the maximum wheel attachment coefficient is generated at an optimum slip ratio. When the driving-wheel slip ratio exceeds this value, the wheel attachment coefficient decreases.

We observe the characteristics of the derivative of wheel attachment coefficient and driving-wheel slip ratio $d\mu_{i}/d\lambda_{i}$ in Fig 5. When $\mu_{i}\leq \mu_{\text{p}i}$, $d\mu_{i}/d\lambda_{i}\geq 0$. The driving wheel is in a stable region and the driving wheel does not slip. When $\mu_{i}>\mu_{\text{p}i}$, $d\mu_{i}/d\lambda_{i}<0$. The driving wheel is in an unstable region and the driving-wheel slips. Notably, $\lambda_{\text{o}i}$ is the driving-wheel optimum slip ratio when $d\mu_{i}/d\lambda_{i}=0$.

$\mu_{i}$ and $\lambda_{i}$ can be obtained as time-series data, and $d\mu_{i}/d\lambda_{i}$ can be calculated from $d\mu_{i}/dt$ and $d\lambda_{i}/dt$ (Eq 18).

$$\frac{d\mu_{i}(\lambda_{i})}{d\lambda_{i}}=\frac{d\mu_{i}}{dt}\cdot \frac{dt}{d\lambda_{i}}$$ (18)

Therefore, once the symbols of the values of $d\mu_{i}/dt$ and $d\lambda_{i}/dt$ are determined, the symbol of $d\mu_{i}/d\lambda_{i}$ is determined.

When EV accelerates, the following rules can be obtained.

(1) The driving wheel is in a stable region. The wheel attachment coefficient increases, and the driving-wheel slip ratio increases.

$$\frac{d\mu_{i}}{dt}>0,\frac{d\lambda_{i}}{dt}>0$$ (19)

(2) The driving wheel is in an unstable region. The wheel attachment coefficient decreases, and the driving-wheel slip ratio increases.

$$\frac{d\mu_{i}}{dt}<0,\frac{d\lambda_{i}}{dt}>0$$ (20)

When EV is decelerating, the following rules can be obtained:

(1) The driving wheel is in a stable region. The wheel attachment coefficient decreases, and the driving-wheel slip ratio decreases.

$$\frac{d\mu_{i}}{dt}<0,\frac{d\lambda_{i}}{dt}<0$$ (21)

(2) The driving wheel is in an unstable region. The wheel attachment coefficient increases, and the driving-wheel slip ratio decreases.

$$\frac{d\mu_{i}}{dt}>0,\frac{d\lambda_{i}}{dt}<0$$ (22)

According to the above analysis, when $d\mu_{i}/dt<0$, the driving wheel runs from the stable region to the unstable region, and the driving-wheel slips. When $\frac{d\mu_{i}}{dt}$ · $\frac{dt}{d\lambda_{i}}>0$, the driving wheel runs from the unstable region to the stable region, and the driving wheel does not slip.

The derivative of the wheel attachment coefficient can be expressed as Eq 23.

$$\frac{d\mu_{i}}{dt}=\frac{1}{N_{i}r_{\text{W}i}}\cdot \frac{d\left(T_{\text{m}i}-J_{\text{W}i}\frac{d\omega_{\text{W}i}}{dt}\right)}{dt}$$ (23)

When a vehicle moves on a road surface, the wheels may turn at a certain angular velocity, but the actual linear velocity of the vehicle may be somewhat lower. This is due to the slippage of the wheels, which does not fully translate to the forward motion of the vehicle. The driving-wheel slip ratio $\lambda_{i}$ is defined as Eq 24. When the vehicle is starting or braking, the slippage of the wheels is more obvious. When the vehicle starts, because $r_{\text{W}i}\omega_{\text{W}i}\geq v_{i}$, the $\lambda_{i}$ is called the acceleration slip ratio. Similarly, when the vehicle is braking, because $r_{\text{W}i}\omega_{\text{W}i}<v_{i}$, $\lambda_{i}$ is called the deceleration slip ratio.

$$\lambda_{i}=\left\{ \begin{array}{c} \frac{r_{\text{W}i}\omega_{\text{W}i}-v_{i}}{r_{\text{W}i}\omega_{\text{W}i}},\;r_{\text{W}i}\omega_{\text{W}i}\geq v_{i}\\ \frac{v_{i}-r_{\text{W}i}\omega_{\text{W}i}}{v_{i}},\;r_{\text{W}i}\omega_{\text{W}i}<v_{i} \end{array}\right.$$ (24)

In this study, we assume that $r_{\text{W}i}\omega_{\text{W}i}\geq v_{i}$ all the time. The derivative of the driving-wheel slip ratio can be expressed as Eq 25.

$$\frac{d\lambda_{i}}{dt}=\frac{v_{i}\frac{d\omega_{\text{W}i}}{dt}-\omega_{\text{W}i}\frac{dv_{i}}{dt}}{r_{\text{W}i}\omega_{\text{W}i}^{2}}$$ (25)

For convenience, *a*_*i*_ and *b*_*i*_ are defined as Eq 26, then Eq 18 can be rewritten as Eq 27.

$$\left\{ \begin{array}{c} a_{i}=\frac{d\left(T_{\text{m}i}-J_{\text{W}i}\frac{d\omega_{\text{W}i}}{dt}\right)}{dt}\\ b_{i}=v_{i}\frac{d\omega_{\text{W}i}}{dt}-\omega_{\text{W}i}\frac{dv_{i}}{dt} \end{array}\right.$$ (26)

$$\frac{d\mu_{i}}{d\lambda_{i}}=\frac{a_{i}}{N_{i}r_{\text{W}i}}\cdot \frac{r_{\text{W}i}\omega_{\text{W}i}^{2}}{b_{i}}$$ (27)

It is evident that when *a*_*i*_ = 0, $d\mu_{i}/d\lambda_{i}=0$. Therefore, we can determine the changes in $T_{\text{m}i}$ and $\omega_{\text{W}i}$ and the driving-wheel optimum slip ratio.

**Fig 6:**
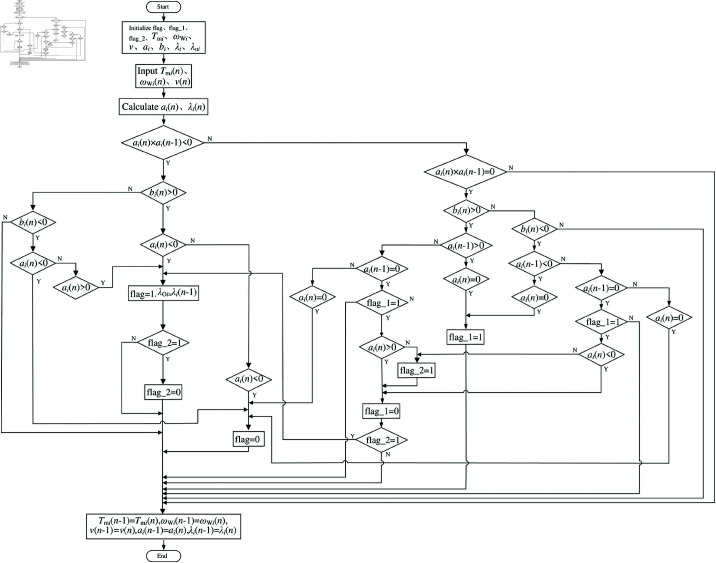
Optimum slip ratio estimation process. https://doi.org/10.6084/m9.figshare.28787756.

When implementing the drive control algorithm, differential calculation is often used to calculate driving-wheel acceleration, so it is easy to produce great oscillation, even when *a*_*i*_ = 0; however, the driving-wheel slip ratio is not large. In addition, accurately capturing *a*_*i*_ = 0 is difficult for several reasons. The optimum slip ratio can be estimated by judging whether the motion state of the driving wheel changes. Literature [47] points out that when the driving wheel moves from the stable region to the unstable region or vice versa, the sign of *a*_*i*_ changes. When we further judge whether the driving wheel is accelerating or decelerating, we can see that the working state of the driving wheel has changed. At this time, we must combine the symbol of *b*_*i*_ to judge. Fig 6 shows the optimum slip ratio estimation process. The acceleration wheel can be determined in practical applications by examining the positive and negative values of *a*_*i*_(*n* − 1) and *a*_*i*_(*n*) before and after the samples. The wheel is considered to initiate slipping during the process of wheel acceleration if *a*_*i*_(*n* − 1)>0 and *a*_*i*_(*n*)<0, and the calculated slip ratio represents the driving-wheel optimum slip ratio of the vehicle under the current driving condition. The optimum slip ratio $\lambda_{\text{o}i}$ for wheel control is directly inherited from the preceding computational iteration, formally defined as: $\lambda_{\text{o}i}=\lambda_{i}(n-1)$ where *λ*_*i*_ (*n* − 1) denotes the slip ratio output of the *i*-th wheel at the (*n*–1)-th control cycle. The slip ratio of the peak adhesion coefficient is determined. This monitoring signal solely pertains to the dynamic parameters of the driving-wheel itself. The determination of whether the wheel has ceased sliding can be made by calculating the truth value of *a*_*i*_ × *b*_*i*_>0 when deceleration occurs. The flag in the figure indicates whether the wheel is operating within the unstable adhesion zone.

## 4 Electronic differential control based on speed and optimum slip ratio estimation

When the vehicle turns, the outer wheel takes a longer path than the inner wheel concurrently, which requires the outer wheel to be faster than the inner wheel, or the external disturbance caused by the uneven road surface causes different resistances on tires when driving in a straight line, which induces differential velocity or tire slip. Therefore, electronic differential control is required.

**Fig 7:**
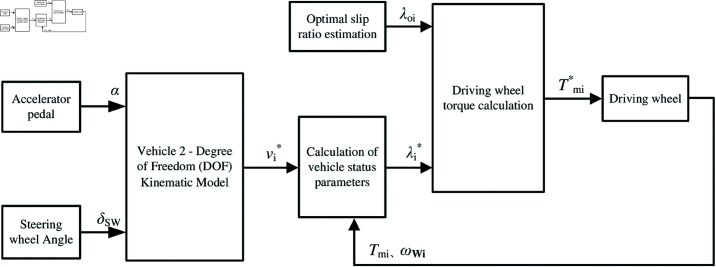
Electronic differential block diagram based on speed and optimum slip ratio estimation. https://doi.org/10.6084/m9.figshare.28787849.

The electronic differential strategies reported in the literature are mainly based on speed and torque. In speed-based electronic differential control, the Ackermann–Jeantand model is often used, through which the wheel velocity is calculated from the steering angle. Because only a static analysis of the motion state is carried out, the influence of tire and centrifugal force is ignored. However, this control strategy has certain limitations. Although some intelligent control methods based on neural networks have been developed since then, non-linear motion control has been fully considered. However, if the vehicle structure changes, the vehicle parameters and mathematical model change accordingly. Parameters need to be adjusted for retraining; therefore, the universality of such strategies is not strong. In addition, when the Ackermann–Jeantand model is used to calculate the wheel velocity, if the steering angle is determined, then the freedom of the four-wheel velocity and vehicle velocity is one. In torque-based electronic differential control, the target value of the driving-wheel slip ratio is controlled according to the state of the vehicle and driving environment based on the vehicle dynamic model. When the tire operates in a stable region, the tire adhesion coefficient is related to the slip ratio and is a monotonic function of the slip ratio. The wheel performs pure rolling motion, and no slip phenomenon occurs. In addition, each wheel is independent of the others and has no restrictions in relation to each other. The motion state of each wheel is determined only by the force of the system. Drag or excessive tire wear between the wheels due to speed mismatch is absent. However, the road conditions of vehicles are often complex and variable, and more random factors affect vehicle driving, which inevitably aggravates the load torque changes for each driving wheel. In this case, if the control variable of the driving wheel is torque, then the electronic differential calculation method on the upper layer of the control system becomes more complicated, with too many dependent variables, and motion coordination control is ignored. Based on the above considerations, this study presents an electronic differential control based on speed and optimum slip ratio estimation for all-electric vehicles with in-wheel motors. Fig 7 shows the frame structure of the controller.

In Fig 7, wheel velocity is first allocated based on the kinematic model. The inner and outer wheels of the vehicle rotate at different speeds around the steering center when turning, ensuring stable vehicle operation with varying strokes within the same time. By combining the vehicle state parameters, one can determine the target initial value of the driving-wheel slip ratio and the target initial value of the driving-wheel driving torque. The driving-wheel slip ratio is then calculated based on the dynamic model. If the driving-wheel slip ratio exceeds the optimum slip ratio estimation, then the driving-wheel slip ratio is equal to the optimum slip ratio estimation. Otherwise, control is performed according to the given initial value of the driving-wheel slip ratio. When $\lambda_{i}\leq \lambda_{\text{o}i}$, the tire is well attached, the driving wheel is in the stable region, and no sliding phenomenon exists. The driver controls the accelerator pedal, given driving torque. When $\lambda_{i}>\lambda_{\text{o}i}$, the driving wheel moves from the stable region to the unstable region. Tire attachment characteristics are reduced, and the sliding occurs. In all-electric vehicles with in-wheel motors, because the driving-wheel torque determines the driving-wheel slip ratio, limiting the driving-wheel torque can control the driving-wheel slip ratio near the optimum slip ratio estimation. The influences of tire force, vertical load change, and vehicle body response are comprehensively considered in this manner. Consequently, the fundamental control structure involves constructing an electronic differential based on the speed control of the 2-DOF kinematic model of the vehicle, coupled with optimal slip ratio control for both left and right wheels, thereby achieving electronic differential control for the rear-wheeled- driven all-electric vehicle.

## 5 Simulation results

**Fig 8:**
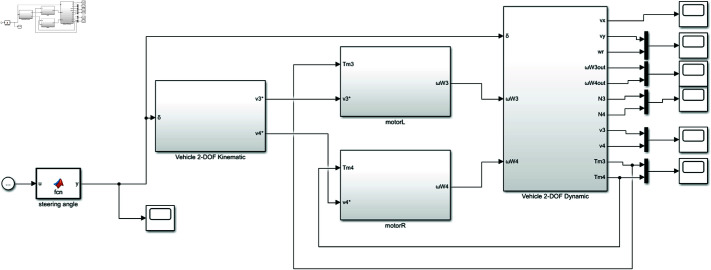
Rear-wheeled-driven all-electric vehicle system model. https://doi.org/10.6084/m9.figshare.28787879.

The rear-wheeled-driven all-electric vehicle system model is depicted in Fig 8, encompassing a steering angle input module, a vehicle 2-DOF kinematic module, two motor controllers, and a vehicle 2-DOF dynamic module.

**Fig 9:**
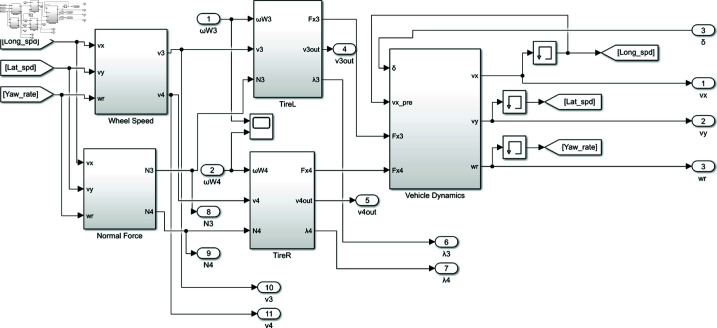
Vehicle 2-DOF dynamics module. https://doi.org/10.6084/m9.figshare.28787891.

The steering angle input module is responsible for modifying the steering angle input and conducting simulation analysis under various operating conditions. By synthesizing the accelerator pedal signal and steering wheel angle signal, the vehicle 2-DOF kinematics module calculates real-time reference velocities for both driving wheels based on current velocity information, which are then fed into the motor controller. The motor controller utilizes a BLDC direct torque control algorithm to achieve rapid and precise motion control of two motors. The vehicle 2-DOF dynamics module primarily consists of two tire stress modules (TirL and TirR), a load calculation module (Normal Force), a wheel speed module, and vehicle body response module (Vehicle Dynamics). It outputs various physical quantities such as longitudinal velocity, lateral velocity, yaw rate, rotational velocities of two wheels, vertical load, and driving torque (as depicted in Fig 9). Parameters of the BLDC motor are listed in Table 1, with vehicle specifications for simulation are shown in Table 2.

**Table 1:** Parameters of the BLDC motor.

Parameters	Value	Parameters	Value
Rated voltage (V)	96	Inductance (mH)	0.85
Rated power (kW)	3	Number of poles	23
Rated speed (r/min)	1000	Torque constant	1.4379
Resistance (Ω)	0.3	Driving wheel yaw inertia (kg · m^2^)	0.15

https://doi.org/10.6084/m9.figshare.28787906

**Table 2:** Vehicle Specifications for Simulation.

Parameters	Value	Parameters	Value
$m_{\text{c}}$ (kg)	800	*B* (m)	1.4
*A* (m^2^)	2	*h* (m)	0.3
$C_{\text{D}}$	0.3	ρ(kg/m^3^)	1.23
*f*	0.018	*g* (m/s^2^)	9.8
rw^(m)^	0.255	$K_{\text{af}}$ (N/rad)	−30000
*l* (m)	2.3	$K_{\text{a}r}$ (N/rad)	−35000
$l_{\text{a}}$ (m)	1.2	$J_{\text{Z}}$ (kg · m^2^)	1050
$l_{\text{b}}$ (m)	1.1		

https://doi.org/10.6084/m9.figshare.28787909

**Fig 10:**
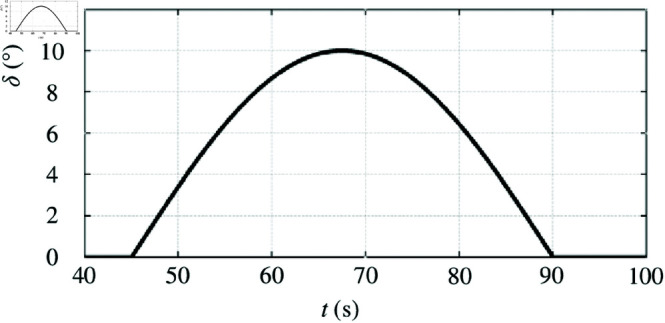
Front wheel steering angle change curve. https://doi.org/10.6084/m9.figshare.28787900.

The steering performance of the electric vehicle was initially validated on a single road surface and simulated at a speed of 40km/h. Assuming a left turn, an optimal slip ratio of 0.3 is considered, with the corresponding change curve for the front wheel steering angle depicted in Fig 10.

**Fig 11:**
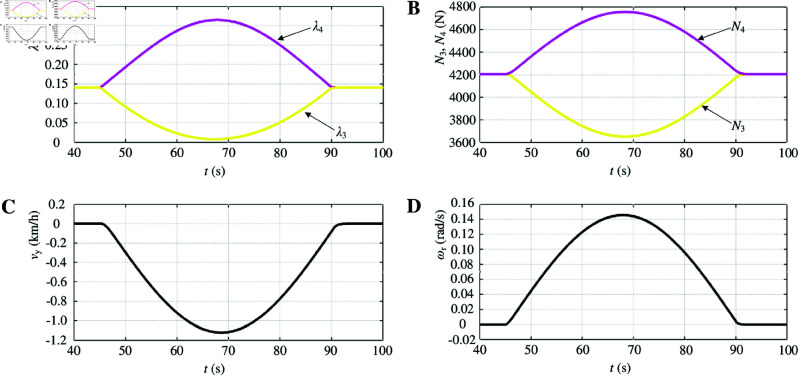
Simulation results of optimum slip ratio control is not included when turning left. A: Driving-wheel slip ratio. B: Driving-wheel vertical force. C: Lateral velocity. D: Yaw rate. https://doi.org/10.6084/m9.figshare.28787918.

The simulation waveform in Fig 11 illustrates the absence of optimum slip ratio control during left turns. As the vehicle steers, the outer wheel’s velocity increases while the inner wheel’s velocity decreases, resulting in changes to both wheels’ slip ratios corresponding to the steering angle variation. When the steering angle becomes sufficiently large, the slip ratio of the outer wheel exceeds 0.3, potentially leading to loss of vehicle control (Fig 11a). During steering, the vehicle body moves in a circular path around the steering center due to centrifugal force, causing an increase in vertical load on the outer wheel and a decrease on the inner wheel (Fig 11b). Fig 11c and 11d represent a graph depicting lateral velocity and yaw rate.

**Fig 12:**
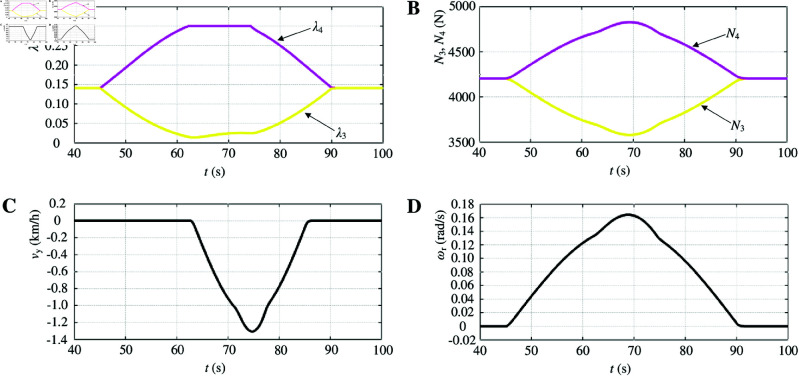
Simulation results of optimum slip ratio control is included when turning left. A: Driving-wheel slip ratio. B: Driving-wheel vertical force. C: Lateral velocity. D: Yaw rate. https://doi.org/10.6084/m9.figshare.28787942.

The simulation waveform in Fig 12 illustrates the inclusion of optimum slip ratio control during left turns. When the vehicle turns, the velocity of the inner wheel decreases while that of the outer wheel increases. As the steering angle increases, the velocity of the inner wheel progressively decreases and that of the outer wheel gradually increases. The slip ratio of the inner wheel diminishes, whereas that of the outer wheel gradually rises. Once a certain angle is reached, typically referred to as a critical value, the slip ratio of the outer wheel reaches its optimal value at 0.3, and it is regulated by a controller to maintain this optimal slip ratio. To maintain the motion posture of the electric vehicle, the slip ratio of the left drive wheel is also curbed (Fig 12a). Similarly, variations can be observed in the driving-wheel vertical force, lateral velocity and yaw rate as well (Fig 12b, 12c, and 12d).

**Fig 13:**
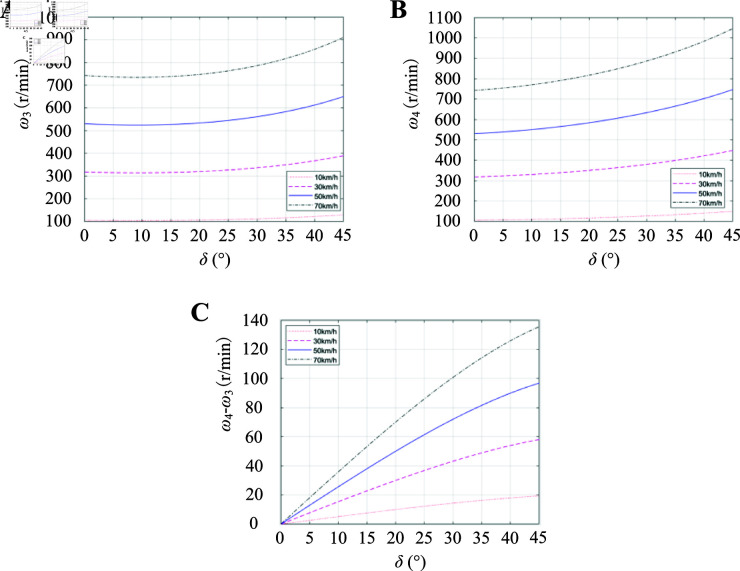
Velocity distribution between $\omega_{3}$ and $\omega_{4}$ and velocity difference between $\omega_{4}$ and $\omega_{3}$. A: $\omega_{3}$ velocity distribution. B: $\omega_{4}$ velocity distribution. C: Velocity difference between $\omega_{4}$ and $\omega_{3}$. https://doi.org/10.6084/m9.figshare.28787954.

**Fig 14:**
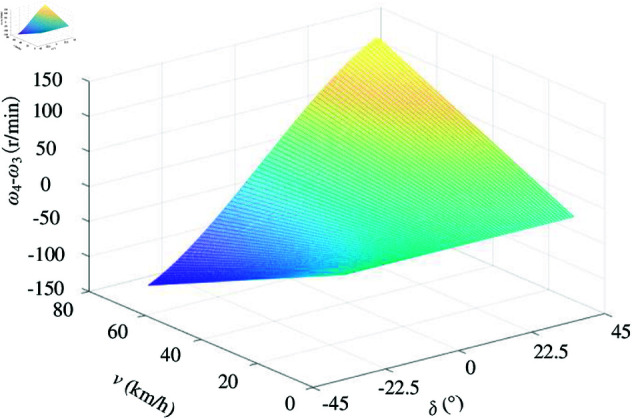
Velocity difference curve with steering angle and vehicle velocity for $\omega_{4}$ and ω_3_. https://doi.org/10.6084/m9.figshare.28787978.

The simulation gradually increases the steering angle from 0° to 45°, considering that the maximum steering angle of the front wheel is 45°. Observation points are set at vehicle velocities of 10km/h, 30km/h, 50km/h, and 70km/h to investigate the velocity distribution of driving wheels 3 and 4 (refer to Fig 13). From Fig 13a and 13b, it can be observed that the inner wheel velocity initially decreases and then increases, while the outer wheel velocity gradually increases with a larger change in velocity as the steering angle increases. In addition, Fig 13c demonstrates that there is an increasing difference between inner and outer wheel velocities as the steering angle increases; moreover, this difference becomes greater with a larger steering angle increase. Fig 14 illustrates how the variation curve of velocity difference between $\omega_{4}$ and $\omega_{3}$ changes with both steering angle and vehicle velocity. When maintaining a constant velocity, there exists a correspondence between front wheel’s steering angle and driving wheel’s rotational velocity. Once determining the specific steering angle value, only one certainty remains: namely, ratio calculation between two-wheel velocities relative to reference velocity can be divided into equal parts for different angles; thus creating tabular data for corresponding ratios of wheel velocities to vehicle velocities using lookup table method combined with interpolation technique simplifies calculations significantly while saving processing time considerably.

In this paper, the electric vehicle driving environment is set within urban road conditions where rain, snow, icy roads, and frequent lane-changing scenarios occur. Consequently, electronic differential control based on speed and optimum slip ratio estimation was further simulated for various cases: joint, μ-split, and lane-change roads. Joint, μ-split, and lane-change road maneuvers are three typical maneuvers used to evaluate the handling and performance characteristics of vehicles effectively. In the simulation, a dry asphalt road offers the best condition, and its maximum wheel attachment coefficient is 1.17. An icy road poses a very poor condition, and its maximum wheel attachment coefficient is 0.19.

(1) Joint road maneuver

**Fig 15:**
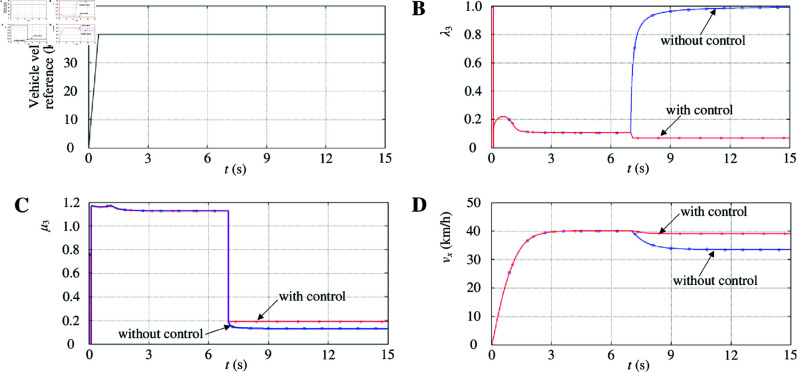
Simulation results with a joint road maneuver. A: Vehicle velocity reference. B: Driving-wheel slip ratio. C: Driving-wheel attachment coefficient. D: Longitudinal velocity. https://doi.org/10.6084/m9.figshare.28788002.

The vehicle velocity reference is shown in Fig 15a. δ is set to 0. The vehicle starts accelerating on a dry asphalt road at an initial velocity of 0 km/h. Then, the vehicle velocity is constant at 40 km/h. At 7 s, the vehicle moves on an icy road.

Evidently, the joint road is symmetrical. At 7 s, the friction force of the left- and right-driving wheels decreases significantly when the vehicle moves on an icy road. However, the trend of the friction force of the two driving wheels is the same. Therefore, the vehicle keeps moving straight. Consider the left-driving wheel here. Fig 15b, 15c, and 15d show the driving-wheel slip ratio, driving-wheel attachment, and longitudinal velocity, respectively. Fig 15b shows the left-driving-wheel slip ratio without control changes in step, and its value changes from 0.1 to above 0.8. The left driving-wheel slips instantly. Fig 15c shows that the left-driving-wheel attachment coefficient without control rapidly decreases and falls below the maximum attachment coefficient of the road, which further reduces the driving capacity of the left-driving-wheel. Although the left driving-wheel slip ratio with control also changes, it quickly stabilizes at the optimum slip ratio. The left driving-wheel attachment coefficient with control quickly returns to a high-attachment state on an icy road, which further increases the driving capacity of the left-driving-wheel. Fig 15d shows that the associated change in the longitudinal velocity with control would not decrease significantly.

(2) μ-split road maneuver

The vehicle velocity reference is shown in Fig 15a. δ is set to 0. The vehicle starts accelerating on a dry asphalt road at an initial velocity of 0 km/h. The vehicle velocity is constant at 40 km/h. At 7 s, the vehicle moves on a μ-split road. The left-driving wheel is still running on a dry asphalt road, while the right-driving wheel is running on an icy road.

**Fig 16:**
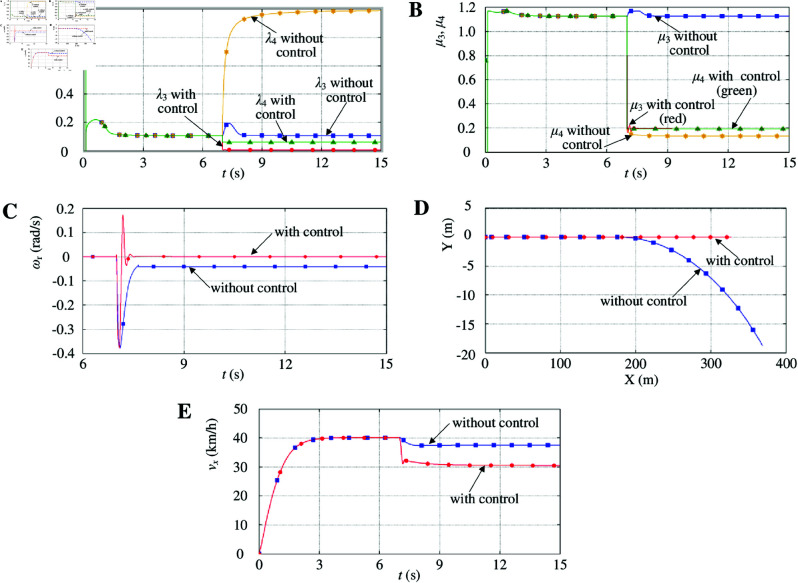
Simulation results with μ-split road maneuver. A: Driving-wheel slip ratio. B: Driving-wheel attachment coefficient. C: Yaw rate. D: Vehicle trajectory. E: Longitudinal velocity. https://doi.org/10.6084/m9.figshare.28788011.

Fig 16a shows the right-driving-wheel slip ratio without control changes in step, and its value changes from 0.1 to above 0.8. The right-driving wheel slips instantly. Although the left-driving-wheel slip ratio without control also changes significantly, it quickly returns to 0.1. Fig 16b shows that the right-driving wheel attachment coefficient without control rapidly decreases and falls below the maximum attachment coefficient of the road, which further reduces the driving capacity of the right-driving wheel. The left driving-wheel attachment coefficient without control quickly returns to a high-attachment state. Fig 16c shows that the yaw rate without control decreases sharply at the beginning because the friction force of the right-driving wheel decreases significantly owing to the μ-split road characteristics. This instantly caused a large yaw moment. As the friction force of the right-driving wheel gradually stabilizes, the yaw rate without control is gradually constant, and finally, a stable yaw moment is produced. Fig 16d shows that a relatively large steering movement is observed, and the vehicle without control cannot maintain straight on. Although the right-driving wheel slip ratio with control also changes immediately, it quickly stabilizes in the optimum slip ratio. The right-driving wheel attachment coefficient with control quickly returns to a high-attachment state on an icy road, further increasing the driving capacity of the right-driving wheel. Concurrently, to enable the vehicle to maintain its course, the left-driving-wheel slip ratio with control also decreases sharply, which further decreases the driving capacity of the left-driving wheel. As a result, the yaw rate with control is effectively controlled. The two driving wheels with control are kept at a synchronous velocity to keep the straight on. Fig 16e shows that the associated change in the longitudinal velocity with control would decrease significantly.

(3) Lane-change road maneuver

**Fig 17:**
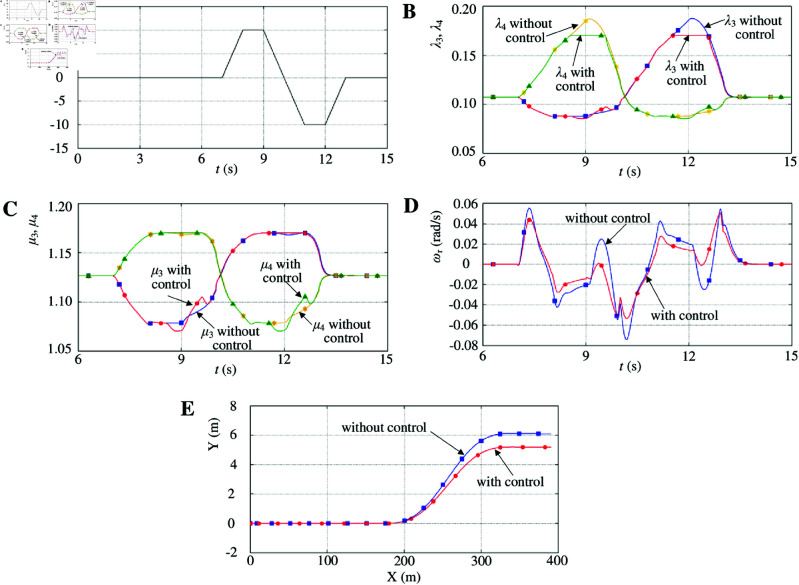
Simulation results with lane-change road maneuver. A: Front-wheel steering angle. B: Driving-wheel slip ratio. C: Driving-wheel attachment coefficient. D: Yaw rate. E: Vehicle trajectory. https://doi.org/10.6084/m9.figshare.28788023.

The vehicle velocity reference is shown in Fig 15a. δ is shown in Fig 17a. The vehicle starts accelerating on a dry asphalt road at an initial velocity of 0 km/h. Then, the vehicle velocity is constant at 40 km/h. At 7 s, the front-wheel steering angle changes. When δ>0, the vehicle turns to the left. When δ<0, the vehicle turns to the right. At 7 s, the vehicle turns to the left. δ is increased linearly from 0° to 10° and held for 1 s, and then decreased linearly from 10° to 0°. At 10 s, the vehicle turns to the right. δ is decreased linearly from 0° to $-10^{\circ }$ and held for 1 s, and then increased linearly from $-10^{\circ }$ to 0°. When δ = 0, the vehicle runs on a straight line.

When the vehicle turns to the left, the right-driving-wheel slip ratio without control gradually increases (Fig 17b). When the front-wheel steering angle is sufficiently large, the right-driving-wheel slip ratio without control exceeds the right-driving wheel optimum slip ratio. The right-driving wheel slips instantly. When the front-wheel steering angle is below a certain value, the right-driving-wheel slip ratio without control gradually decreases, and the right-driving wheel gradually no longer slips. When δ>0, the left-driving-wheel slip ratio without control varies with the front-wheel steering angle; however, the left-driving wheel optimum slip ratio surpasses that of the left-driving-wheel slip ratio without control. The left-driving wheel does not slip. When the vehicle turns to the left, the right-driving wheel attachment coefficient without control gradually decreases (Fig 17c). The right-driving wheel gradually slips, which further decreases the driving capacity of the right-driving wheel. When δ>0, the left-driving wheel attachment coefficient without control varies with the front-wheel steering angle, but the left-driving wheel attachment coefficient without control is always in a high-attachment state. Although the right-driving-wheel slip ratio with control also gradually increases, it quickly stabilizes in the right-driving wheel optimum slip ratio when the right-driving-wheel slip ratio exceeds the right-driving wheel optimum slip ratio. The right-driving wheel attachment coefficient with control quickly returns to a high-attachment state, which further increases the driving capacity of the right-driving wheel. Simultaneously, to ensure that the vehicle maintains its course, the left-driving-wheel slip ratio with control also varies with the front-wheel steering angle, which further suppresses the driving capacity of the left-driving wheel. Fig 17d shows that the yaw rate with control is effectively controlled. When the vehicle turns to the right, the changes in the driving-wheel slip ratio, the driving-wheel attachment coefficient, and the yaw rate are similar to its left turn, which will not be repeated. Fig 17e shows that the vehicle trajectory with control decreases significantly.

In order to validate the effectiveness of the proposed electronic differential control strategy based on speed and optimum slip ratio estimation, the maximum transmissible torque estimation (MTTE) control strategy was selected as the comparative research object. The MTTE control strategy estimates the instantaneous friction between the wheel and the ground by utilizing the wheel acceleration and the electromagnetic torque of the motor, thereby calculating the maximum driving force that can be provided between the tire and the ground [48]. During the startup of a all-electric vehicle with in-wheel motors, slippage may occur due to the abrupt increase in motor torque and wheel velocity. Consequently, a comparative simulation analysis of the vehicle’s starting condition was conducted first. The simulation conditions were set as follows: (1) When the driver presses the accelerator pedal within 0.5 s, a linear acceleration torque command with a slope of 0.5 is generated, assuming the vehicle is operating on a high-quality road surface with an adhesion coefficient of 0.9; (2) The vehicle skid ceases after 2 s.

**Fig 18:**
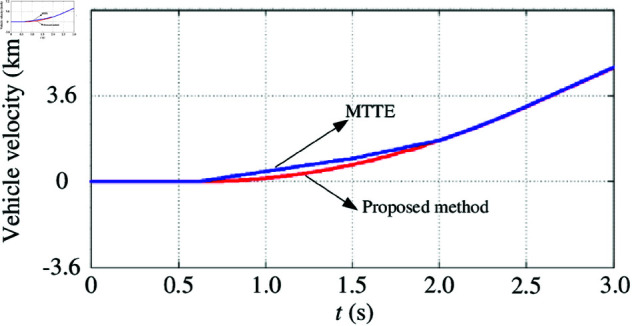
Vehicle starting velocity under two control strategies. https://doi.org/10.6084/m9.figshare.28788029.

Fig 18 demonstrates that under the electronic differential control based on speed and optimum slip ratio estimation, the wheel slip induced by starting torque can be effectively suppressed during vehicle startup. This approach ensures a more stable initiation of motion and a seamless transition to regular driving conditions.

Next, the acceleration performance of the two control strategies is validated under sliding road surface conditions. The simulation setup is as follows: (1) The driver operates the vehicle with a specified torque (T^*^ = 200 N·m) and performs linear acceleration on a road surface exhibiting sliding characteristics. Under normal road conditions, the reference driving torque for each of the two driving wheels is half of the given torque, so the initial reference torque for each wheel is T^*^ = 100 N·m. (2) From 0 to 5 s, the road adhesion coefficient is μ=0.9. (3) From 5 to 5.5 s, the road adhesion coefficient decreases to μ=0.3. (4) After 5.5 s, the road adhesion coefficient recovers to μ=0.9.

**Fig 19:**
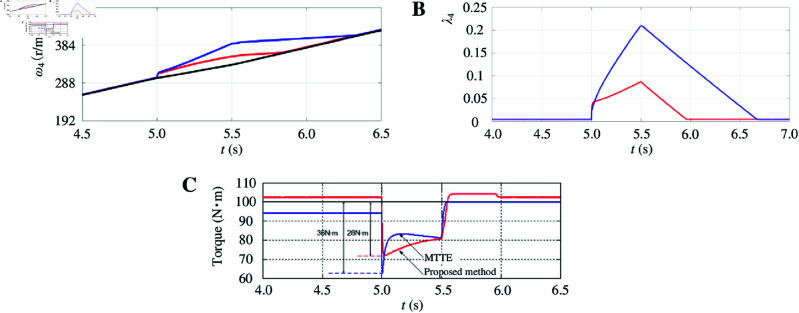
Acceleration performance of the two control strategies under sliding road surface conditions. A: Right rear driving wheel angular velocity. B: Right rear driving wheel driving torque. C: Right rear driving wheel slip ratio. https://doi.org/10.6084/m9.figshare.28788047.

The following discussion uses the right rear wheel (driving wheel 4) as an example. As shown in Fig 19, the vehicle was in the acceleration phase until 5 s. Given that the road adhesion coefficient μ=0.9 indicates a high-quality road surface, no wheel slippage occurred, and both control strategies effectively met the operational requirements of the vehicle. However, electronic differential control based on speed and optimum slip ratio estimation generated relatively lower driving forces. Between 5 and 5.5 s, the vehicle entered a skid-prone road surface, causing the wheels to slip. As depicted in Fig 19a, under both control strategies, the wheel speed increased significantly. Notably, the electronic differential control based on speed and optimum slip ratio estimation exhibited a greater increase in wheel velocity and a higher corresponding slip ratio (see Fig 19b), which approached the optimum slip ratio, thereby minimizing the loss of driving wheel driving torque (see Fig 19c). From Fig 19b, it is evident that although the slip ratios under both control strategies remained within the range of the optimum slip ratio during the adjustment process, the electronic differential control based on speed and optimum slip ratio estimation more closely approximated the optimum slip ratio, resulting in a smaller reduction in total driving force and superior control performance.

## 6 Experimental results

**Fig 20:**
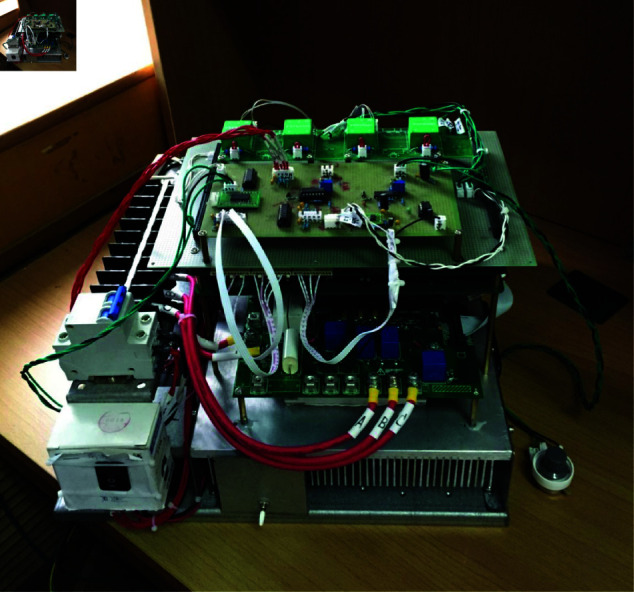
Vehicle controller prototype. https://doi.org/10.6084/m9.figshare.28788056.

The vehicle controller prototype embraces an integrated design philosophy, amalgamating two motor controllers and a differential controller into a singular vehicle controller, as illustrated in Fig 20. The TMS320F28335 chip serves as the core control unit. The external circuitry primarily encompasses critical components like the three-phase inverter bridge, isolated drive circuitry, position detection circuitry, and current detection circuitry. Notably, the three-phase inverter bridge undertakes the crucial role of power conversion and regulation. The isolated drive circuitry ensures efficient electrical isolation and steady operation among distinct circuit segments. The position detection circuitry is capable of acquiring real-time and precise position information, while the current detection circuitry facilitates the detection of current data. The PWM switching frequency of the inverter is set at 20kHz.

**Fig 21:**
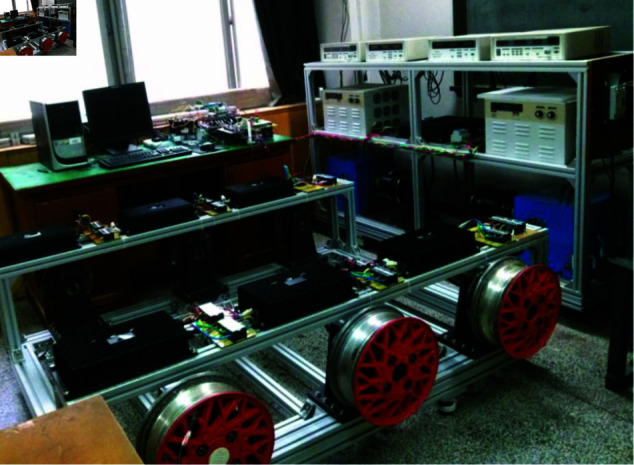
Test bench designed for specialized vehicles driven by 8 × 8 in-wheel motors. https://doi.org/10.6084/m9.figshare.28788065.

Due to the limited availability of research funding, the authors currently lack the necessary resources to conduct sample vehicle testing. To validate the static performance of the motor controller and the differential control capability of the two drive wheels, experiments were performed on the test bench for a specialized vehicle propelled by an 8 × 8 in-wheel motors. Through this test bench, the coordination control capability between the central controller and each motor controller was verified, ensuring that each motor complies with the specified speed requirements. Furthermore, the experimental setup provides a critical reference for subsequent prototype vehicle testing and demonstrates significant practical value. The results indicate that the functionality of the motor controller and the differential control of drive wheel 3 and drive wheel 4 meet the required specifications. The test bench for the 8 × 8 in-wheel motor-driven specialized vehicle comprises a DC power supply, 8 permanent magnet brushless DC motors, a dynamometer system assembly, a central controller, and 8 motor controllers. The central controller utilizes the TMS320F28335 chip as its control core, supplemented by peripheral circuits such as CAN bus communication interface. Fig 21 illustrates the test bench designed for specialized vehicles driven by 8×8 in-wheel motors.

**Fig 22:**
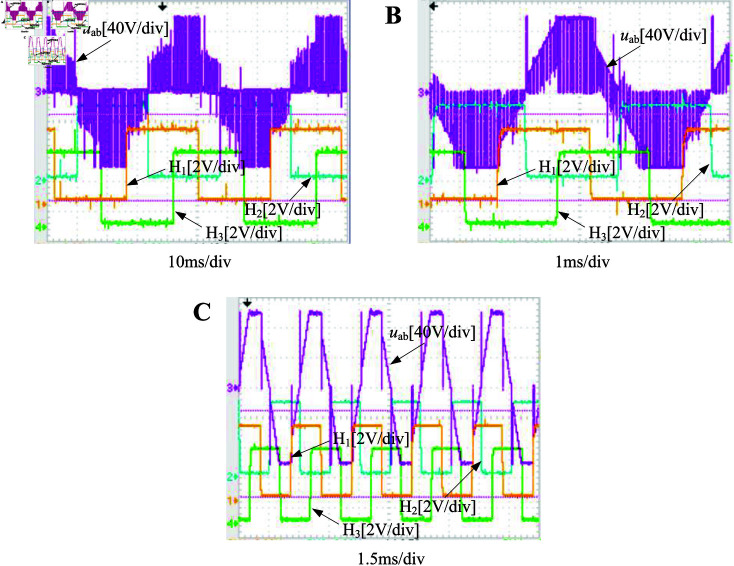
Driving wheel output waveform under different velocity. A: 50 rpm. B: 450 rpm. C: 900 rpm. (${\text{H}}_{1}$, ${\text{H}}_{2}$, and ${\text{H}}_{3}$ represent the Hall sensor signal, while *u*_*ab*_ denotes the voltage of the ab line.) https://doi.org/10.6084/m9.figshare.28788074.

**Fig 23:**
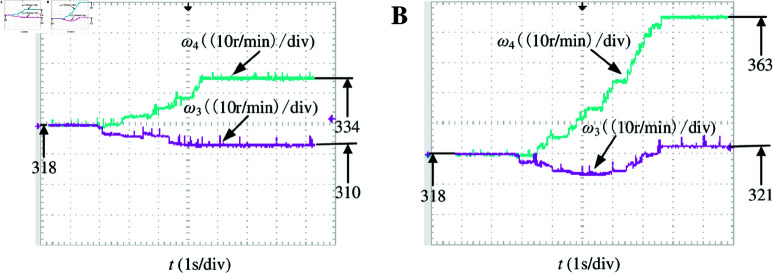
Velocity waveform of each driving wheel under different steering conditions. A: *v* = 30km/h, δ = 10°. B: *v* = 30km/h, δ = 20°. https://doi.org/10.6084/m9.figshare.28788086.

The output waveforms of the driving wheel at different velocities are illustrated in Fig 22, demonstrating the motor’s stable operation and compliance with design requirements. Fig 23 examines the differential control effect of driving wheels 3 and 4 under varying steering angles at a constant velocity, revealing their ability to allocate velocity based on differential strategy.

## 7 Conclusion

The electronic differential control based on speed solely analyzes the static motion state, disregarding the non-linear characteristics of tires and other influencing factors, thereby exhibiting significant limitations. The motion state of the electronic differential control based on torque is exclusively determined by the system forces, neglecting motion coordination control. The load torque of each driving wheel fluctuates frequently, leading to increased complexity in the control system. Due to the constraints imposed by the electronic differential strategy, which utilizes speed and torque as control variables, we present a method for improving the driving performance of two driving wheels subjected to uneven external disturbances on the road surface. This approach integrates the benefits of electronic differentials control based on speed and torque, aiming to address the issue of extreme environmental adaptability of electric vehicles under typical operating conditions, with a focus on vehicle stability to improve driving safety. We mainly studied electronic differential control based on speed and optimum slip ratio estimation, with the aim of maintaining the driver’s maneuvering ability at the maximum adhesion limit. Under urban road conditions, natural phenomena such as rain, snow, and ice on the road, along with frequent lane changes during driving, necessitate the selection of joint road manoeuvre, μ-split road manoeuvre, and lane-change road manoeuvre. The proposed method can improve the safety of all-electric vehicles with in-wheel motors under critical driving conditions, as evidenced by the simulation and experimental results, in which the performance of the proposed method was validated. And establish the groundwork for the subsequent vehicle testing. Additionally, based on the identified limitations and potential opportunities in the research, future work will focus on three key directions to enhance the adaptability and safety of electric vehicles under complex environmental conditions. (1) By integrating real-time optimum slip ratio estimation with adaptive tire dynamics models, the performance of the control strategy under extreme conditions (e.g., black ice, gravel, or flooded surfaces) will be optimized, thereby significantly improving the system’s robustness in rapidly changing environments. (2) A hierarchical control architecture will be developed to dynamically balance competing objectives such as energy efficiency, driving wheel driving torque, and vehicle stability, ensuring an optimal trade-off and overall performance. (3) Leveraging hardware-in-the-loop (HIL) systems and prototype vehicle platforms, large-scale real-world vehicle tests will be conducted based on existing simulation and preliminary experimental results to validate the computational efficiency and reliability of the proposed control strategy under real-time constraints.

## Nomenclature

$m_{\text{c}}$: Vehicle mass.
$J_{\text{z}}$: Vehicle yaw inertia.
l: Wheel base.
$l_{\text{a}}$: Distance from center of gravity to front axle.
$l_{\text{b}}$: Distance from center of gravity to rear axle.
B: Distance between wheels.
h: Height of center of gravity.
$v_{\text{a}}$: Vehicle velocity.
$v_{i}$: Wheel velocity, *i*=1, 2, 3, 4.
$v_{x}$: Longitudinal velocity.
$v_{y}$: Lateral velocity.
$\omega_{\text{r}}$: Yaw rate.
δ: Steering angle.
r: Steering radius.
$r_{i}$: Wheel steering radius, *i*=1, 2, 3, 4.
β: Sideslip angle.
$\alpha_{i}$: Wheel sideslip angle, *i*=1, 2, 3, 4.
$\delta_{i}$: Wheel steering angle, *i*=1, 2, 3, 4.
$\eta_{i}$: Track angle of the wheel, *i*=1, 2, 3, 4.
$F_{xi}$: Wheel longitudinal force, *i*=1, 2, 3, 4.
$F_{yi}$: Wheel lateral force, *i*=1, 2, 3, 4.
$\mu_{i}$: Wheel attachment coefficient, *i*=1, 2, 3, 4.
$F_{zi}$: Wheel vertical load, *i*=1, 2, 3, 4.
$K_{\text{a}i}$: Wheel Cornering Stiffness, *i*=1, 2, 3, 4.
$T_{\text{m}i}$: Driving wheel driving torque, *i*=3, 4.
$F_{\text{d}i}$: Driving wheel friction force, *i*=3, 4.
$N_{i}$: Driving wheel vertical force, *i*=3, 4.
$J_{\text{W}i}$: Driving wheel inertia, *i*=3, 4.
$\omega_{\text{W}i}$: Driving wheel angular velocity, *i*=3, 4.
$r_{\text{W}i}$: Driving wheel radius, *i*=3, 4.
$\lambda_{i}$: Driving wheel slip ratio, *i*=3, 4.
